# Assessment of Skeletal Muscle Contractile Properties by Radial Displacement: The Case for Tensiomyography

**DOI:** 10.1007/s40279-018-0912-6

**Published:** 2018-03-31

**Authors:** Lewis J. Macgregor, Angus M. Hunter, Claudio Orizio, Malcolm M. Fairweather, Massimiliano Ditroilo

**Affiliations:** 1 Faculty of Health Sciences and Sport, University of Stirling, Stirling, Scotland UK; 2 Dipartimento di Scienze Cliniche e Sperimentali, Università degli Studi di Brescia, Brescia, Italy; 3 SportScotland Institute of Sport, Stirling, Scotland UK; 4 School of Public Health, Physiotherapy and Sports Science, University College Dublin, Dublin, Ireland

## Abstract

Skeletal muscle operates as a near-constant volume system; as such muscle shortening during contraction is transversely linked to radial deformation. Therefore, to assess contractile properties of skeletal muscle, radial displacement can be evoked and measured. Mechanomyography measures muscle radial displacement and during the last 20 years, tensiomyography has become the most commonly used and widely reported technique among the various methodologies of mechanomyography. Tensiomyography has been demonstrated to reliably measure peak radial displacement during evoked muscle twitch, as well as muscle twitch speed. A number of parameters can be extracted from the tensiomyography displacement/time curve and the most commonly used and reliable appear to be peak radial displacement and contraction time. The latter has been described as a valid non-invasive means of characterising skeletal muscle, based on fibre-type composition. Over recent years, applications of tensiomyography measurement within sport and exercise have appeared, with applications relating to injury, recovery and performance. Within the present review, we evaluate the perceived strengths and weaknesses of tensiomyography with regard to its efficacy within applied sports medicine settings. We also highlight future tensiomyography areas that require further investigation. Therefore, the purpose of this review is to critically examine the existing evidence surrounding tensiomyography as a tool within the field of sports medicine.

## Key Points

**Table:** 

Tensiomyography is a technique that measures radial deformation of skeletal muscle, and in turn its contractile properties, following a stimulated muscle contraction. It resembles the more established mechanomyography process and in fact it should be regarded as a special case of mechanomyography, with some advantages and disadvantages vs. comparable methods.
Peak radial displacement and contraction time are the two most common parameters extracted from the displacement/time curve as a result of a single-twitch stimulus. Contraction velocity combines peak radial displacement and contraction time and appears to provide information on twitch rate without being affected by changes in peak radial displacement.
In recent years, tensiomyography has been used in a number of applications, such as non-invasive estimation of muscle fibre composition, determination of muscle stiffness, adaptation to training in sporting populations, fatigue, muscle damage and recovery, and bilateral muscle asymmetries
Construct validity and reliability of tensiomyography have been established; however, not for specialist populations (elite athletes, clinical patients). The issue of the low level of muscle contraction elicited raises questions over the external validity of the technique for some applications.

## Introduction

Muscle contraction occurs as a result of electro-mechanical coupling that determines shortening of sarcomeres and in turn the whole muscle fibre. Simultaneous contraction of thousands of muscle fibres causes shortening of the long axis of a muscle [1]. It has been proposed, as early as the 17th century by Swammerdam [2], that the skeletal muscle operates as a near-constant volume system, therefore shortening of the long axis must be linked to an increased transverse diameter [3]. More recently, relationships between muscle motor output and its geometry changes have been measured by using a real-time, brightness mode ultrasonic apparatus to follow instantaneous changes in gastrocnemius muscle fascicle length [4]. Simultaneous recording of torque alongside geometry changes provides evidence that tension is generated synchronously with changes in fascicle length. It is also established that surface mechanomyography (MMG) can measure the expansion of muscle fibres during contraction [4]; a strict relationship exists between the MMG amplitude and the oscillations of the output torque and the fascicle length. At a macro level, there are overlapping dynamics between torque oscillations at tendon- and surface-detected MMG, during both stimulated and voluntary contractions [5, 6]. This further supports the concept that the muscle can be regarded as a near-constant volume system, with muscle fibre shortening and thickening, as detected by muscle surface displacement and tension at the tendon level [3]. In conclusion, muscle surface displacement can be a useful tool to track muscle contractile features during contraction. This method may be useful to establish the status of the muscle for either sporting performance or rehabilitation.

Therefore, this review starts by providing an overview of how the radial displacement of the skeletal muscle is evoked and measured to assess its contractile properties. Mechanomyography is briefly introduced as the most established method to measure muscle radial displacement. Thereafter, a more recent method, tensiomyography (TMG), which measures radial displacement following electrical stimulation of the muscle, is the main focus of this review. Tensiomyography uses the same principle as MMG; however, it is designed to work only with stimulated muscle contraction and uses a unique mechanical sensor to detect muscle radial displacement. We discuss the validity, reliability and applications of TMG and examine the existing evidence surrounding TMG as a practical tool within the field of sports medicine, highlighting strengths and weaknesses. Finally, a case is made that TMG can be regarded as a special case of MMG.

### Literature Search

We searched the scientific literature relevant to this review between January and July 2017, using the US National Library of Medicine (PubMed) and the Google Scholar database. The terms ‘tensiomyography’, ‘TMG’, ‘radial displacement’, ‘maximal displacement amplitude’, ‘contraction time’, ‘delay time’, ‘sustain time’ and ‘half relaxation time’ were used in different combinations to retrieve pertinent articles. We also sourced relevant literature from the reference list of articles obtained from the database searches. Most TMG applications were reviewed as detailed in Sect. 4. A few TMG articles dealt with clinical applications outside the sports and exercise medicine field and, in keeping with the scope of Sports Medicine, they were not included. A total number of 227 articles were retrieved and 55 TMG papers were included. Only full-text articles and books were used for this review; congress abstracts are not included.

## Assessment of Muscle Contractile Properties

### Evoked Muscular Contraction

Muscle contraction can be voluntary or stimulated. Whilst the MMG technique has been used with either type of contraction, TMG only operates with stimulated muscle contraction. Therefore, in this review we only refer to stimulated muscle contraction.

Single twitch can be defined as the contractile response to a single electrical impulse and is a specific type of evoked muscle activity used to characterise the mechanical properties of a muscle or a single motor unit. The electrical stimulus can be applied to the motor nerve [7] or to the motor point identified on the muscle surface [8]. Either way, the stimulation travels through the nerve fibre and eventually reaches the muscle fibre. A rectangular stimulus is the most used in practice; however, stimuli of different shapes can be delivered [9]. Additionally, stimuli should be bipolar, with a negative area trailing the positive one, to avoid the polarization at the electrode–skin interface. Polarization may result in a less efficient current injection through the nerve. The duration of the stimulus spans from 25 to 1000 µs in subjects without neuromuscular diseases.

In the last 30 years, a number of studies have recorded the MMG signal following single-twitch stimulation. Early work of Barry [10] and Frangioni et al. [11] described the mechanisms of MMG generation in isolated frog muscle. A more recent paper by Kaczmarek et al. [12] examined in more detail the outward and inward muscle surface displacement and has related this phenomenon to the orientation of the muscle fibres. The time-relationship between electrical activation of the muscle, muscle surface displacement and tension generation has been studied by means of MMG in several studies, revealing a shorter latency of the MMG signal compared to the electromyogram activity [e.g. 13–15].

Mechanomyography detected by piezoelectric [16] or electric condenser [17] microphones, during evoked ‘single motor unit’ activity, produces a signal amplitude presenting a linear relationship with the specific rate and amount of force. Results also are dependent on the fast or slow classification of the investigated unit. A similar conclusion has been reported when a “whole muscle” response is analysed in slow- and fast-twitch human muscles [18]. These early experiments could be considered as the underpinning evidence that muscle surface displacement is strictly linked to features of muscle mechanical output at the tendon; hence, they provide the rationale for the TMG technique application reviewed here.

Changes in muscle contractile properties associated with peripheral fatigue have been extensively studied in the time domain analysis of evoked single twitch. In this condition, the amplitude of recorded MMG parallels observed torque reduction [15, 19, 20]. However, when twitch amplitude increases because of post-activation potentiation, the recorded MMG [21] and TMG [22] amplitudes also increase, which may [23] or may not [24] be accompanied by increased twitch speed. Specific neuromuscular diseases show a lengthening of the electromechanical delay [25] or a depression of the post-activation potentiation, [26] and this has been reflected by a reduction in the MMG signal amplitude.

Evoked muscular contraction using single-twitch stimulation, with the TMG signal being recorded, has been explored in a number of studies and applications. This evidence is extensively covered from Sect. 3 onwards.

### Measurement of Radial Displacement of the Skeletal Muscle Following a Stimulus

The usefulness of detecting a change in muscle thickness to characterise muscle contraction was first recognised almost 100 years ago. In a textbook of physiology from the early 20th century, it is advocated that “… the analysis of the mechanical effects of muscle excitements would be incomplete if only changes in muscle length were examined disregarding changes in muscle thickness …” [27]. About 50 years later, Margaria and De Caro [28] detailed a method to record the contraction wave from the changes in muscle thickness. A curarized muscle was directly electrically stimulated by two wires, and two levers transduced the surface displacement on a kymograph.

In more recent years, the technology for detecting radial displacement of skeletal muscle has obviously improved thanks to the development of a variety of relatively lightweight and low-cost sensors. The sensitivity of the sensors has also improved, along with the ability to process and analyse the signal. This has made this type of measurement more common. The laser sensor can detect the distance between the laser-beam head and the surface of the muscle and therefore any change in the distance as a result of muscle contraction [29]. Very light accelerometers secured to the skin have also been used for this purpose, either a single piezoelectric accelerometer [e.g. 30] or an array of accelerometers to characterise the spatial activity distribution of the muscle [31]. Alternatively, an accelerometer mounted on a probe pressed against the muscle has been used [32]. Piezoelectric contact sensors and microphones have been mainly used during dynamic muscle contractions [33]. The contact sensors are mechanically coupled to the muscle surface (using elastic or adhesive bands or an external support), whereas the microphones are coupled to the muscle surface via air, ultrasound gel or surgical cement [29].

Laser sensors, accelerometers, contact sensors and microphones have been used in the past 30 years to assess muscle function using MMG. An alternative sensor to detect radial displacement of skeletal muscle was proposed for the first time in 1996 [34]. However, a comprehensive description of this sensor only appeared the following year—it consisted of a spring-loaded probe embedded in a displacement sensor and pressed against the muscle with a pressure of 0.2 N/cm^2^ [35]. Interestingly, even though the latter is commonly regarded as the first study to have employed TMG, the term was not used. To the best of our knowledge, it was only in 2001 that a study was published using a very similar sensor (probe with a pressure of 0.015 N/mm^2^) and the term TMG was adopted for the first time [36]. A recent study made the first direct comparison between a contact sensor (similar to that employed in TMG) and a laser sensor [37]. After five consecutive single-twitch maximal stimulations, either sensor measured muscle displacement of the same portion of the muscle belly. Both MMG sensors showed good to excellent test–retest reliability. However, when comparing them a systematic bias was observed, with the contact sensor recording greater values than the laser sensor. The authors attributed this difference to the nature of the sensor (the laser is not in direct contact with the skin, unlike the contact sensor). They also argued that the differences may be clinically irrelevant.

## Tensiomyography: A Special Case of Mechanomyography?

### Tensiomyography: Overview of the Technique

Over the last two decades, primarily through pioneering work at the Faculty of Electrical Engineering, University of Ljubljana, Slovenia, [35, 36, 38–40] TMG has been developed as an alternative method to measure radial deformation of muscle. A high-precision (4-µm) digital displacement sensor is applied to the muscle belly with a controlled pre-tension between the sensor tip and the muscle. It is this pre-tension from which the method has derived the name TMG [36], and by providing controlled pre-tension the muscle twitch response is augmented, enhancing the measurement of contraction dynamics [41].

**Fig. 1:**
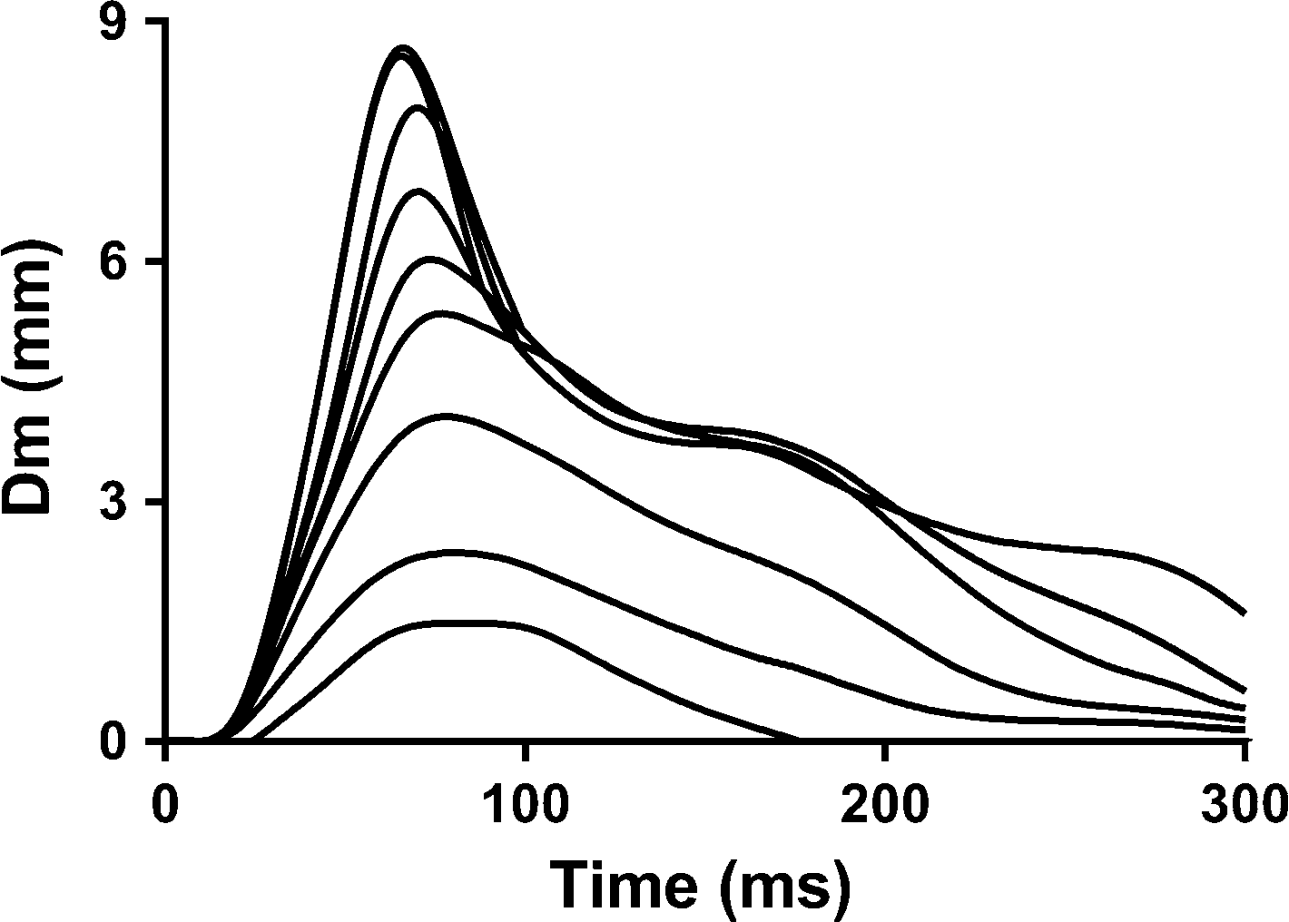
Typical incremental progression of displacement curves. The increase in curve magnitude is induced by an increase in stimulation amplitude (typically up to 60–100 mA). Peak displacement (Dm) is identified by a plateau in displacement curves, despite increased stimulation amplitude. Peak radial displacement signifies the absolute spatial transverse deformation of the muscle

Muscle twitch is induced through a single 1-ms-wide electrical stimulus. In the TMG literature, there is currently no consensus regarding the polar orientation of electrodes. Indeed, this information has often been absent; however, we recommend the cathode be placed proximal to the anode, in line with the details provided in Sect. 2.1. Stimulation amplitude is variable inter-individually, as the amplitude required to provide a maximal muscle response is not equal among all muscles or individuals. It would be inappropriate to apply a similar stimulation amplitude universally as multiple factors influence muscle response, including: motor unit recruitment threshold, skin conductivity, subcutaneous depth, water retention and temperature [41]. Typically, studies report peak responses occurring at stimulation amplitudes between 60 and 100 mA. To identify the maximal required stimulation amplitude, and thus peak muscle response, a progressive incremental approach is adopted [42]. Stimulation amplitudes of increasing intensity are delivered intermittently—a time interval of 10 s is allotted between consecutive measurements to restrict the impact of fatigue and potentiation on the muscle under investigation [41]. Peak muscle twitch is identified by a plateau in displacement curves that, despite an increased stimulation amplitude, does not result in greater muscle displacement (Fig. 1).

Care must be taken to ensure precise positioning of sensors and electrodes, to avoid the detection of co-activation of deeper or neighbouring muscles, which could be misconstrued as further increases in peak radial displacement (Dm). An initial amplitude of 20–30 mA, with 10-mA incremental increases (up to a maximum of 110 mA), has been most widely adopted. Increments of < 10 mA may lead to an observed plateau, before supra-maximal stimulation has been reached. A small number of peer-reviewed studies have adopted a different approach, using a single universal amplitude (typically 100 mA); whilst this may provide adequate results, the rigor of such an approach is less than that of the incremental protocol. The incremental approach allows the operator to visualise the development of the twitch curve, as it grows towards a plateau with increasing stimulation amplitude (Fig. 1); this is not possible with the single stimulation method. We therefore recommend following the incremental protocol, which has been most widely reported within published research to date.

**Fig. 2:**
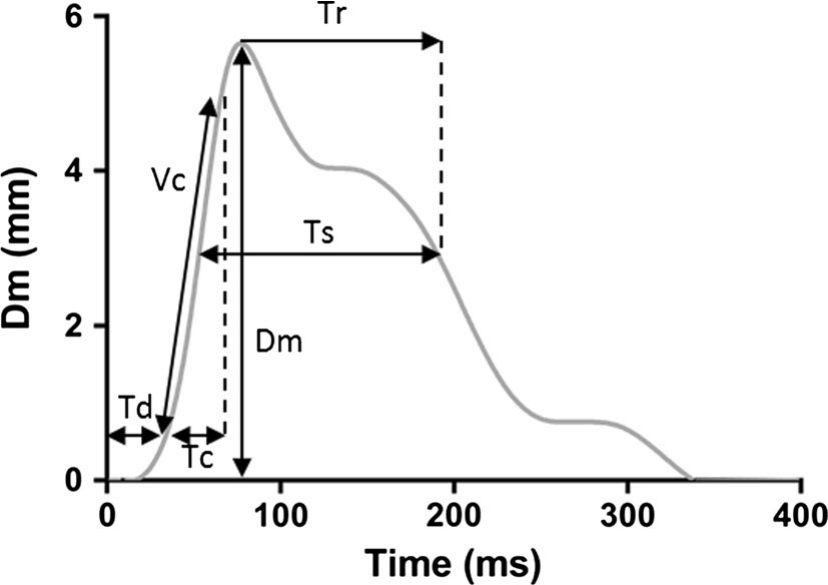
Parameters extracted from a typical displacement curve; displacement (Dm), contraction time (Tc), delay time (Td), contraction velocity (Vc) [Vc = (90%Dm − 10%Dm)/Tc], sustain time (Ts) and half-relaxation time (Tr)

A number of parameters are extracted from twitch displacement/time curves (Fig. 2). Peak radial displacement signifies the absolute spatial transverse deformation of the muscle; reduced Dm is interpreted as an increase in muscle stiffness, therefore larger Dm implies lower muscle stiffness [43]. Contraction time (Tc) is measured as the time taken on the ascending curve between 10 and 90% of Dm. Contraction time therefore reflects the speed of twitch force generation; longer Tc means a slower twitch force generation, which could be a reflection of muscle fibre type but it could also depend on decreased tendon stiffness [43]. Among children aged 9–14 years, regular participation in sport (at least 3 h/week in the previous 5 years) has been associated with shorter Tc of biceps femoris [44]. In a similar population, shorter Tc of biceps femoris has been associated with faster running speed [45]. Delay time (Td) represents the time between delivery of the electrical stimulus and 10% of Dm, providing a measure of muscle responsiveness [46]. Half-relaxation time is given as the time taken from 90 to 50% of Dm on the descending curve; the duration for which twitch is sustained (Ts) is measured as the time between 50% of Dm on each side of the twitch curve, with each of these latter two parameters providing a theoretical assessment of muscle fibre fatigue status [46, 47].

Contraction time is partly dependent on Dm, i.e. the greater Dm is the longer the Tc will be, if muscle excitability is the same. Therefore, it is important to measure the actual velocity of the muscle contraction. Contraction velocity (Vc) can be calculated as the rate of ΔDm between 10 and 90% relative to Tc [48]. Whilst Tc provides a measure of the speed at which the muscle reaches maximal Dm, Vc describes the rate of muscle contraction, and is independent of Dm. Similarly, Valenčič and Knez, [35] Rodríguez-Ruiz et al. [49] and Rodríguez-Ruiz et al. [50] have all reported normalised response speed (Vrn), representing the relationship between ΔDm between 10 and 90% and ΔTc divided by absolute Dm. It is not uncommon for Tc and Dm to alter disproportionately to one another; however, in such instances, we propose that the change in Tc, independent from Dm, will be driven by an alteration in the rate of contraction, as measured by Vc or Vrn. The most appropriate method to define Vc has yet to be established, and a number of variations have been described. In addition to the approach presented above, Loturco et al. [51] calculated Vc by dividing Dm by the sum of Tc and Td. Others have calculated Vc from the time elapsed when Dm had reached a specific threshold: 2 mm, [52, 53] or 10 and 90% of Dm [54]. We suggest that further study is required to establish the most appropriate standard for assessing twitch velocity in relation to objective functional markers of muscle speed.

### Validity and Reliability of Tensiomyography

Given that TMG is intended to measure, in vivo, contractile and mechanical properties of the skeletal muscle, criterion-referenced validity is inherently difficult to quantify. The construct validity of the TMG technique has been determined in a number of papers by relating the Tc, or Vc, to the proportion of slow-twitch fibres of the muscle/s examined (Table 1). The very first attempt was conducted 20 years ago [35]. The authors showed, in one single subject, that Vc was lower in muscles known for having a higher percentage of slow-twitch fibres and was higher in faster muscles. Similarly, Valenčič et al. [42] related Tc measures to the percentage of type I muscle fibres provided from mammalian reference material (reported by Ariano et al. [55]) and reported an *R*^2^ = 0.81. A slightly stronger research design was used a few years later [36]; muscle biopsies were taken from a group of 15 cadavers of healthy individuals who died suddenly. The percentage of type I muscle fibres was found to be positively correlated with Tc (*r* = 0.93), as measured by TMG, in a group of 15 subjects, matched with the cadavers for age and physical characteristics.

**Table 1:** Summary of the studies correlating contraction time (Tc), one of the tensiomyography parameters, with and slow-twitch muscle fibre type

Study, year	Comparison	Correlation
Valenčič et al. [42]	Coefficient of determination: Tc vs. type I fibre %	0.81
Dahmane et al. [36]	Pearson’s correlation coefficient: Tc vs. type I fibre %	0.93
Dahmane et al. [38]	Pearson’s correlation coefficient: Tc at 50% of supramaximal response stimulation vs. deep region type I fibre %	0.90
Šimunič et al. [56]	Pearson’s correlation coefficient: Tc vs. MHC-1%	0.88

*Tc* contraction time, *MHC* myosin heavy chain

In a follow-up study using the same two samples, [38] the muscle fibre type was identified for the superficial and deep portion of a number of muscles in the cadaver group. The 15 subjects were measured for TMG using low- and high-intensity electrical stimulation, with the intention of recruiting superficial and deep portions of the muscle, respectively. The authors found a positive correlation between Tc at low-intensity stimulation and the percentage of type I muscle fibres in the superficial portion of the muscle (*r* = 0.76); and a positive correlation between Tc at high-intensity stimulation and the percentage of type I muscle fibres in the deep portion of the muscle (*r* = 0.90). A recent study [56] adopted a more robust research design than any of the previous investigations. A larger sample size was used (*n* = 27) and from the same subjects, TMG measurements and muscle biopsies from the vastus lateralis were taken. The authors found a positive and significant correlation between the proportion of myosin heavy chain I and Tc (*r* = 0.88), Td (*r* = 0.61) and Tr (*r* = 0.67).

In an attempt to contribute to construct the validity of TMG using a different approach, Dahmane et al. [39] observed a difference in Tc of the biceps femoris between 15 healthy men (30.25 ± 3.5 ms) and 15 male sprinters (19.5 ± 2.5 ms) aged 17–40 years. An inverse and significant correlation between Tc and running speed in sprinters was also reported (*r* = − 0.72, *p* < 0.05).

Reliability of TMG measurement parameters has previously been investigated across a variety of conditions, in a number of studies (Table 2). Krizaj et al. [41] analysed short-term repeatability of each parameter extracted from muscle twitch responses, by delivering 30 consecutive stimulations and capturing individual measurements from each twitch response. Intra-class correlation coefficient (ICC) of 0.86 or greater was recorded for all parameters. Inter-rater reliability has also been examined, with specific focus on the importance of transducer and electrode positioning [47]. In a similar finding, ICC was greater than 0.86 for each parameter, and the coefficient of variation (CV) below 5%, with the exception of Tr. It was noted that deliberately altering the inter-electrode distance significantly impacted the measurement of Dm, likely owing to a change in the recruitment pattern. Muscle fibres do not shorten homogeneously, owing to non-uniform sarcomeres, myofibres or fascicle lengths [57]. Therefore, consideration is also necessary regarding sensor positioning as morphological changes may occur in a muscle when the sensor is repositioned [58].

**Table 2:** Tensiomyography (TMG) reliability

Study, year	Muscle		TMG parameters				
			Dm	Tc	Td	Ts	Tr
Krizaj et al. [41]	Biceps brachii	ICC	0.98	0.97	0.94	0.89	0.86
		CV (%)	x	x	x	x	x
Tous-Fajardo et al. [47]	Vastus medialis	ICC	0.97	0.92	0.86	0.96	0.77
		CV (%)	4.7	3.4	2.7	14.2	2.4
Šimunič [59]	Vastus lateralis	ICC	0.99	0.98	0.89	0.96	0.89
		CV (%)	1.8	1.5	4.4	7.6	4.7
Ditroilo et al. [60]	Gastrocnemius medialis	ICC	0.86 to − 0.95	0.62 to − 0.92	0.56 to − 0.62	0.71 to − 0.86	0.67 to − 0.79
		CV (%)	8.0 to − 14.8	3.8 to − 9.4	7.0 to − 9.2	5.3 to − 8.2	27.8 to − 32.7

*x* not measured, *TMG* tensiomyography, *ICC* Intra-class correlation coefficient, *CV* coefficient of variation

*Dm* displacement, *ICC* intra-class correlation coefficient, *Tc* contraction time, *Td* delay time, *Ts* sustain time, *Tr* half-relaxation time, *Ts* sustain time

Inter-day reliability has returned comparatively favourable results, [59] with Dm and Tc in particular displaying ICCs of no less than 0.98, and CVs below 5%, across three separate lower limb muscles. However, the practical applicability of these findings must be queried, as measurements were performed following 60 min of bed rest. As such, Ditroilo et al. [60] evaluated the reliability of TMG in different muscle conditions—rested, exercised and fatigued; additionally assessing long-term (4-week interval) stability of the contractile parameters. Although reliability was reported to suffer when measurements were carried out over longer intervals (ICC ranging from 0.86 to 0.95 and CV from 14.8 to 8.0% for Dm; ICC ranging from 0.62 to 0.92 and CV from 9.4 to 3.8% for Tc), it is interesting that the measurements displayed greater reliability in an exercised or fatigued state, compared with a rested state.

As a result of the reported findings of these four reliability studies, it has been recommended that assessment carried out through TMG should focus on the most stable parameters, namely Dm and Tc [61]. Half-relaxation time is consistently the least reliable parameter across studies; in intra-session reliability (ICC = 0.86 [41]); in inter-session reliability (CV ranging from 6.4 to 9.3%, ICC < 0.90 [59]); in inter-rater reliability (CV = 14.2%, ICC = 0.77 [47]); and in long-term stability (CV ranging from 29.4 to 32.7%, ICC < 0.82 [60]).

In accordance with recommendations derived from the above-mentioned reliability studies, [41, 47, 59, 60] Dm and Tc have been the most thoroughly investigated parameters using TMG. The existing body of research suggests that TMG may provide a valid alternative to more invasive analyses of skeletal muscle properties [32]. Peak radial displacement is typically considered in association with muscle stiffness and tendon mechanical properties [56, 62–64]; although to date, no study has compared Dm with a direct assessment of muscle stiffness in vivo. It has also been shown that fluctuations in muscle size, such as following disuse atrophy, [43, 65] are associated (*r* = − 0.70) with changes in Dm; thus increased Dm accompanied a reduction in muscle thickness. This decreased thickness would have led to diminished muscle stiffness, [66] allowing greater Dm in response to electrical stimulation.

To apply TMG effectively, it is important to integrate TMG parameter measurements with physiologically functional variables. That said, the relationship between contractile parameters (Dm and Tc) and muscle function has yet to be fully elucidated. Comparisons between twitch torque/force and TMG-recorded parameters [67, 68] have suggested different mechanisms affect longitudinal and transversal skeletal muscle deformations. Torque, exerted by a contracted muscle belly, and detected longitudinally, must transmit via connective tissue to be measured externally, with a degree of slack present in the musculotendinous unit, which must be taken up before the connective tissue exerts the torque that represents muscle belly twitch force. Additional damping occurs from passive surrounding tissue, further interfering with longitudinal twitch interpretation [67]. Measuring temporal features of muscle radial displacement may provide limited insight into contraction speed. Because Tc is extracted as the duration of twitch between 10 and 90% of peak Dm, Tc is highly dependent on the size of Dm. Therefore, Tc may not provide an objective assessment of Vc. As such, to date, Dm, potentially assessing excitability [54] and/or passive tension, [56, 62–64] would appear to be the most advantageous mechanical measurement using TMG. Relative assessments of the speed of Dm (Vc and Vrn) may prove to be insightful, [69]; however, further investigations under different physiological conditions are required.

## Applications of Tensiomyography

### Assessment of Sporting Populations

As highlighted in the previous section, the validity and reliability of TMG have not been well established among specialist populations, including elite athletes. That being said, the use of TMG within a sports setting has recently been discussed [70]. In particular, interest surrounds the intensive demands of training and competition. Where supercompensation [71] transitions towards functional overreaching, performance may be negatively affected [72]. Muscle mechanical adaptations have been measured through TMG with strength [52, 54] and endurance training [46]. Additionally, differences in mechanical properties between endurance and strength/power athletes have been described [73]. Measurements of Dm were lower following high-load resistance exercise compared with workload-matched high-volume training [52]; while Tc was longer, and Dm tended to be reduced, following 6 days of high-intensity interval training [70].

Among the few sport-specific applications of TMG assessment published to date, soccer has received the greatest attention. Soccer studies focussing on injury have described how reconstructive surgery and subsequent rehabilitation, following anterior cruciate ligament injury, have resulted in modified contractile mechanics of muscles in lower extremities on the injured limb, compared with both the contralateral limb and to uninjured soccer players [74]. Furthermore, Alentorn-Geli et al. [75, 76] have proposed TMG as an appropriate screening tool to investigate knee flexor muscle stiffness as a risk factor for anterior cruciate ligament injury, with injured individuals presenting greater Dm and longer Tc (in the uninjured limb) compared with injury-free individuals [75]. The authors suggested that these differences, between individuals who have experienced an anterior cruciate ligament injury and those with no history of an anterior cruciate ligament injury, could hint at elevated susceptibility to ligament injury. Balance between contractile mechanics of knee flexors and extensors may be linked to co-contraction and this was also suggested as an important predictor for injury risk [75].

Such studies have strengthened the case for inclusion of TMG muscle contractile assessment within training [77] and rehabilitation programmes [78]. We suggest that further integration of TMG with established physiological markers is required to validate the technique for use further within high-performance sport. Soccer has also been the basis for longitudinal research into contractile alterations across a 10-week training cycle [79]. Spatial and temporal parameters were influenced by the training period, with reported reductions in Dm and Tc of knee extensors; furthermore, lower Dm in knee extensors and flexors were observed among soccer players compared with a control group. Similarly, García-García et al. [63] presented data describing longer Tc in latissimus dorsi, and longer Tc and Td, as well as larger Dm, in the trapezius, among female kayakers compared with non-kayakers. These differences are attributed to kayak-specific training. Differences have also been described between Vrn of knee extensors and flexors among volleyball players of different positions [50].

Greater insight could be provided by incorporating more regular measurements, throughout training cycles, as well as overlapping mechanical assessments with functional performance measures [e.g. running speed, counter movement jump (CMJ)]. To this end, Gil et al. [80] examined the association between TMG parameters and performance indicators (tests of jumping and sprinting) in elite soccer players. A moderate association (*r* ≈ 0.5; *p* < 0.05) was only found between Dm and parameters linked to the stretch–shortening cycle performance. This further supports the idea of Dm being a reflection of muscle stiffness [43]. In a similar type of approach, using physiological performance indicators, García-García [81] related TMG parameters to maximal oxygen consumption and power at maximal oxygen consumption and found a significant correlation between Dm and these performance indicators (*r* > 0.6; *p* < 0.05) only for biarticular muscles such as the biceps and rectus femoris. However, the study does not seem to provide a convincing physiological explanation for the observed association.

While the studies discussed in this section provide a promising start, there is certainly a demand for further evidence to grow our understanding of the direct links between TMG-derived twitch parameters and muscle performance. It would be remiss not to question the external validity of the technique within sporting applications given the low level of muscle contraction elicited; adding context to the parameters is crucial, and incorporating well-understood physiological measures in parallel with TMG is undoubtedly the sensible approach. Moreover, intervention-based research will be invaluable as the use of TMG continues to expand into the domain of elite sport.

### Muscle Fatigue and Exercise-Induced Muscle Damage

To date, ten papers have been published examining the use of TMG to monitor the effect of fatigue. However, because the types of fatigue that can be induced are quite different, we have divided the studies into general vs. local fatigue (Table 3). General fatigue was induced using an ultra-endurance triathlon, [46] an uphill-marathon, [82] lower limb strength training, [54] a 6-day high-intensity interval training, [70] a 6-day strength training, [83] or a 6-day strength or endurance training micro-cycle [69]. Local fatigue was induced using two different protocols of arm curls, [52] 2 min of cycling at maximal aerobic capacity [84] or a 5-min electrical stimulation programme [48].

**Table 3:** Tensiomyography (TMG) response after fatigue

Study, year	Type of fatigue/EIMD	TMG parameters		
		Dm	Tc	Vc
Wiewelhove et al. [86]	General	=	=	=
Raeder et al. [83]	General	↓	=	x
de Paula Simola et al. [69]	General	↓	=	↓
de Paula Simola et al. [54]	General	↓	↓	↓
Wiewelhove et al. [70]	General	↓ (trend)	↑	x
Giovanelli et al. [82]	General	↑	↓	x
García-Manso et al. [46]	General	↑	↑	x
Macgregor et al. [48]	Local	↓	x	=
García-Manso et al. [52]	Local	↓	x	↓
Carrasco et al. [84]	Local	↓	x	x
Hunter et al. [89]	EIMD	↓	↑	x

↑ increased; ↓ decreased; = unchanged, *x* not measured. *TMG* tensiomyography, *EIMD* exercise induced muscle damage, *Dm* displacement, *EIMD* exercise induced muscle damage, *Tc*  contraction time, *Vc* contraction velocity

As expected, a decline in Dm was observed in the three studies that induced local fatigue [48, 52, 84]. This occurrence was likely owing to impaired propagation of the electrical stimulus along the sarcolemma, resulting in less muscle displacement. This impairment is likely to occur from a pH-driven alteration of the Na^+^ and K^+^ gradient [85] across the muscle membrane, resulting in reduced Ca^2+^ and subsequent excitation–contraction coupling, [48] or through accumulation of inorganic phosphate within muscle cells.

Interestingly, of the seven studies inducing general fatigue, four reported a decrease in Dm, [70, 83] two showed an increase in Dm, [46, 82] whilst the other showed no change [86]. It has to be noted though that Raeder et al. [83] Wiewelhove et al. [70] de Paula Simola et al. [54] and de Paula Simola et al. [69] all used variations of high-intensity resistance or endurance training, or interval training, over short periods of time. Conversely, two other studies established the effects of ultra-endurance on TMG, namely an Ironman^®^ triathlon [46] and an uphill-marathon (43 km, 3063 m elevation gain; [82]). Wiewelhove et al. [86] also adopted a high-intensity interval protocol, but despite reporting a decline in CMJ, there were no alterations in Dm. It is worth noting that CMJ incorporates multiple muscle groups across multiple joints, as well as including a central component, [87] while TMG analyses individual muscles in isolation from the central nervous system; for this reason, careful contextual consideration is required before attempting to assess muscle fatigue through TMG alone. In the case of the reduced muscle stiffness observed following ultra-endurance running, [46] it may follow that elevated cytokine release stimulated altered peripheral feedback [88]. Furthermore, conflicting results were reported for Tc, which showed a post-fatigue increase [46, 70] or decrease, [82] or no change [83].

As discussed in Sect. 3, changes in Tc should be treated with caution, as measurements are subject to biasing influences from changes in Dm. Vc and Vrn are relative measures of twitch contraction speed, and therefore remain independent from Dm; however, Macgregor et al. [48] and Wiewelhove et al. [86] both reported no change in Vc in fatigued muscle. Furthermore, de Paula Simola et al. [54] and de Paula Simola et al. [69] both demonstrated decreases in Vc; interestingly, impaired Vc was delayed until 72 h after completion of high-intensity endurance training, unlike high-intensity strength training, following which Vc was impaired immediately.

One previous study [89] adopted TMG as an objective assessment of exercise-induced muscle damage. Peak radial displacement was reduced, while Tc was extended, following eccentric exercise. Changes in Dm followed the same profile as reductions in muscle force capacity [correlation coefficient ranged from *r* = 0.55 (± 0.2) to *r* = 0.67 (± 0.27)], with a strong relationship also between Dm and creatine kinase levels, and changes in limb circumference and muscle soreness. It seems that TMG Dm can provide useful insights when assessing fatigue or muscle damage, although temporal parameters should be treated with caution.

### Recovery

It is important to incorporate routine assessment of fatigue and recovery into training programmes, to help inform optimisation of training prescription and ensure competition readiness. Practically however, individuals will respond variably to given training stimuli, hence inconsistencies in quantifiable fatigue or recovery markers are commonplace [70]. Exorbitant levels of intense training, particularly incorporating only brief recovery time, subject the musculoskeletal system to considerable physiological demands, impairing subsequent performance [90]. Two different modalities of recovery have been investigated. The first is active recovery, which features submaximal ‘warm-down’ exercises combined with stretching target muscles [91]; it is designed to promote greater peripheral blood flow, preventing venous pooling, and to attenuate symptoms of muscle soreness and reduce musculotendinous stiffness. Passive recovery involves no specialised activities, but instead relies on resting for a period of time to promote muscle restoration. Rey et al. [92] compared 12 min of low-intensity running and 8 min of lower limb static stretching, with 20 min of passive recovery, in professional soccer players; complete recovery of Td, Tc and Dm was observed in the biceps femoris and rectus femoris, following both interventions. Recovery using whole-body vibrations following high-intensity exercise did not cause a change in the TMG variables when compared to passive recovery [84]. Similarly, no effect of foam rolling, a sports recovery tool, was observed on the TMG variables [93]. Following passive recovery only, elevated muscle soreness was reported, but despite this, there was no difference in TMG parameters between active and passive recoveries.

Comparing passive recovery following high-load or high-volume resistance training, García-Manso et al. [52] observed a more rapid recovery of biceps brachii Dm following high-volume (i.e. lower load) training between 6 and 15 min following the completion of exercise. Half-relaxation time differed between groups, with high-load training resulting in longer Tr than high-volume training between 6 and 10 min following exercise; Tr was similar between groups by 15 min post-exercise. Sustain time also was longer following high-load training, but only up to 6 min post-exercise.

In another study, García-Manso et al. [94] described reductions in Dm, as well as slower Vc, following exposure to cold water immersion, a common practice to help recovery after high-intensity sport activity. The authors attributed this alteration in muscle stiffness to reduced Ca2 + transport and changes in viscoelastic properties of the muscle. Based on the existing evidence, TMG can provide a non-invasive assessment of exercise recovery and will not in itself impact the recovery process.

### Muscle Fibre Type

Five studies have attempted to relate the parameters extracted from TMG to type I muscle fibres to explore whether TMG can be used as a non-invasive method to estimate muscle fibre-type composition. These studies also represent the foundation for determining the construct validity of the TMG technique and have been described in detail in Sect. 3.2. Table 1 summarises the main results.

Very recently, Zubac and Šimunič [95] applied a non-invasive estimation of type I muscle fibres to link measurements of Dm and Tc to a performance marker (CMJ), following 8 weeks of plyometric training. They demonstrated an inverse correlation (*r* = − 0.67) between improved CMJ following training and a change in estimated myosin heavy chain I.

### Muscle Stiffness

While TMG will detect alterations in rates of muscle contraction, it will also measure muscle passive tension attributed to connective tissue elements between muscle fibres along with sarcoplasm and sarcolemma [96]. However, there have been a limited number of TMG studies that have experimentally isolated muscle length changes to determine how effective TMG is for measuring muscle stiffness per se. Pišot et al. [43] measured TMG and muscle thickness loss following 35 days of bed rest whereupon they demonstrated increased Dm alongside reduced muscle thickness. This reduced muscle mass would have reduced muscle stiffness to allow for greater Dm in response to the stimulus [66]. This negative correlation between muscle thickness and Dm indicates a lower muscle resting tension, resulting from muscle atrophy. As such, TMG amplitude (Dm) reflects muscle belly stiffness in the same way that MMG amplitude has been suggested to do [97].

An alternative method for altering stiffness of a muscle is to change its length by flexing the joint it is attached to. Ditroilo et al. [98] did this by flexing the knee joint and measuring TMG of the biceps femoris at three different angles (0°, 45° and 90°); as expected Dm increased alongside joint angle. Nevertheless, when using TMG to detect muscle status it can be difficult to differentiate between excitation and contraction coupling and stiffness changes, as in most situations both parameters are affected. Koren et al. [68] provide evidence that temporal parameters extracted by TMG from the displacement curve are shorter compared with the same parameters extracted from the twitch torque curve, suggesting that they are related more to intrinsic muscle properties. As such, TMG provides a complementary measure of muscle contraction dynamics to typical measurements of force and torque responses.

Currently, there is a shortage of data investigating potential intervention-associated alterations in muscle stiffness, using TMG. One case study has been published [99] describing increased Dm associated with reduced muscle stiffness, as a result of a dry needling treatment in a stroke patient. We propose that the application of TMG as a measure of interventions designed to alter local muscular stiffness is an area that requires greater attention.

### Symmetry

Bilateral muscular asymmetry has previously been investigated in association with injury prevalence [100] and more recently, athletic ability [101, 102]. Indeed, bilateral strength asymmetry can predict injury [103] and sporting performance [104, 105]. Monitoring of asymmetry has also been conducted to assess the effectiveness of rehabilitation programmes and to inform when an individual is able to return to their sport or activity [106]. Tensiomyography allows for assessment of asymmetry at the individual muscle level, allowing the specific muscle underlying any bilateral imbalance to be identified [74–76]. Asymmetric TMG responses have been reported in injured soccer players, prior to anterior cruciate ligament reconstruction, with greater symmetry restored following surgery [74].

However, in a different study, asymmetries have been reported to persist for more than 2 years following surgery [107]. Uninjured soccer players were assessed for symmetry, with no differences between limbs observed for biceps femoris or rectus femoris, in Dm or Tc [74, 80]. Vastus lateralis and vastus medialis did, however, display asymmetry with regard to Tc [74]. A study with a similar population showed less than 10% bilateral asymmetry in vastus medialis and up to about 20% asymmetry in the vastus lateralis, rectus femoris and biceps femoris [108]. In another study, volleyball players have also shown high levels of displacement symmetry in lower limb muscles, regardless of sex, which is in contrast to the untrained general population [50]. Temporal assessment of muscle twitch, however, revealed asymmetric differences in contraction speed in the vastus lateralis in men, and in the vastus medialis, rectus femoris and biceps femoris in women. These observations are attributed to the specific series of skills that are trained in sports such as volleyball [50]. To reduce asymmetry-associated injury risk, TMG could provide a useful measure, by identifying specific muscles that are causing the asymmetry, which additionally is feasible during injury rehabilitation.

## Perceived Strengths and Weaknesses of Tensiomyography

There is a general consensus among practitioners that the TMG device is relatively inexpensive compared with laboratory-based equipment, easy to carry and set up, with a quick data collection process. This makes TMG ideal to be used in a field setting, in particular for sports applications, such as monitoring training load and fatigue. Not only is the data collection quick and easy, the pre-tension sensor tip produces a favourable signal-to-noise ratio in muscle response, avoiding the need for special filtering or post-processing. This makes the TMG technique attractive for sports medicine practitioners and conditioning coaches. Additionally, TMG isolates the muscle of interest, excluding confounding central variables that could bias measurement. Owing to methodological requirements discussed above, such as sensor and electrode positioning, it is suggested that appropriate training and expertise should be applied to capture high-quality data.

One of the main limitations of TMG is that the device only allows the examination of electrically stimulated contractions. The level of contraction elicited appears to be fairly low. Valenčič et al. [42] concurrently measured Dm and torque of the tibialis anterior muscle in healthy young subjects and reported values of about 0.3–3.0 mm and 0.1–1.1 Nm, respectively. The same subjects recorded tibialis anterior isometric torque of above 15 Nm during a slow isometric contraction up to the maximum. Ditroilo et al. [98] reported that the level of knee flexor torque recorded following an electrically stimulated contraction using TMG was lower than 10% of maximal voluntary contraction, which has been confirmed by Maffiuletti as reported in Ditroilo et al. [98] and briefly mentioned by Šimunič et al. [56] Surprisingly, this issue has received very little attention in the literature; however, it questions the external validity of the technique, especially for applications to sports performance. Before a conclusion on external validity can be reached, ad-hoc studies examining the relationship between electrical stimulation and level of torque elicited should be conducted on different muscle groups. Interestingly, Pišot et al. [43] managed to isolate the digital displacement sensor of the TMG and apply a voluntary contraction. They found that the muscle displacement increases linearly with muscle torque up to 68% of maximal voluntary contraction and it levels off afterwards. The muscle examined is not known though, thus this again would need more investigation in an attempt to gain a better insight into the relationship between the stimulus and response provided by TMG. As such, there is still much investigation required to pinpoint direct links between TMG and muscle function.

## Conclusions

Tensiomyography is an in vivo non-invasive method to examine mechanical and contractile properties of the skeletal muscle. In previous sections, similarities and differences between TMG and MMG have been highlighted; they both use an electrically stimulated contraction to record the radial displacement of the muscle. In TMG, this is done using a digital displacement sensor. Even though the TMG manufacturer and most of the authors of TMG studies regard this technique as novel and unique, with the evidence we have provided we advocate that TMG is a special case of MMG. This might imply a terminology change or clarification in future TMG publications, and a credible consensus statement would help this.

Our final comments for researchers and practitioners are:

Even though construct validity and reliability of TMG have been ascertained, the issue of the low level of muscle contraction elicited questions on the external validity of the technique, at least for some of the applications.

Tensiomyography has been used in a number of applications, most of which concern sports performance and muscle contraction properties.

Reliability and validity among specialist populations (elite athletes, clinical patients) have not been described; therefore, before progressing with the application of TMG in such specialist populations, further research should be carried out to establish its efficacy.

Traditionally, five parameters are extracted from the TMG curve. However, the two most used are Dm and Tc, which are also the parameters with the highest level of reliability; Dm is a valid measure of muscle stiffness, while Tc can predict muscle fibre-type composition. The use of Vc, which combines Dm and Tc, is increasing.
